# Exploratory Diagnostic Performance of Salivary Cortisol for Initial Assessment of Recent Exposure to Interpersonal Physical Violence: A Preliminary Study

**DOI:** 10.3390/diagnostics16193138

**Published:** 2026-09-27

**Authors:** Szymon Rzepczyk, Kacper Nijakowski, Jakub Jankowski, Artur Teżyk, Karina Sommerfeld-Klatta, Paweł Świderski, Natalia Macioszek, Klaudia Dolińska-Kaczmarek, Czesław Żaba

**Affiliations:** 1 Department of Forensic Medicine, Poznan University of Medical Sciences, 60-806 Poznan, Poland; 2 Department of Conservative Dentistry, Poznan University of Medical Sciences, 60-812 Poznan, Poland; 3 Doctoral School, Poznan University of Medical Sciences, 60-812 Poznan, Poland; 4 Department of Toxicology, Poznan University of Medical Sciences, 60-806 Poznan, Poland

**Keywords:** saliva, violence, cortisol, hormones, biomarkers

## Abstract

**Background/Objectives**: Enhancing the objectivity of the assessment of interpersonal physical violence has become a challenge in modern forensic sciences. The use of biomarker assessments, such as cortisol, has become a promising diagnostic tool. Moreover, this hormone can be assessed non-invasively by measuring its concentration in saliva. This preliminary study evaluated salivary cortisol concentrations following recent interpersonal physical violence and assessed their apparent exploratory diagnostic performance for distinguishing violence-exposed individuals from controls. **Methods**: A study group (n = 26) was recruited from individuals referred by the Police for forensic medical examination within 48 h of the violent incident. Samples were taken between 8:30 and 10:30 a.m. A medical interview and physical examination preceded sample collection. Saliva was collected using the passive drool method. Samples were centrifuged, and morning salivary cortisol concentrations were determined using ELISA. The control group (n = 26) was recruited concurrently from healthy volunteers. **Results**: Salivary cortisol concentrations were approximately 4.7-fold higher in individuals exposed to physical violence than in matched controls (110.33 ± 54.41 vs. 23.49 ± 6.29 ng/mL; *p* < 0.001), and this difference remained significant after adjustment for age, sex, BMI, and sample collection time (*p* < 0.001). ROC analysis demonstrated near-perfect apparent discrimination between violence-exposed individuals and controls, with an AUC of 0.991 (95% CI: 0.962–1.000; *p* < 0.0001). The optimal cut-off of >35.45 ng/mL yielded a sensitivity of 92.31% (95% CI: 74.9–99.1%) and specificity of 100% (95% CI: 86.8–100.0%). Bootstrap analysis yielded a 95% CI for the optimal cut-off ranging from >32.17 to >35.45 ng/mL. **Conclusions**: Salivary cortisol demonstrated strong apparent discriminatory performance for recent exposure to interpersonal physical violence in this preliminary cohort. However, the proposed threshold should be regarded as exploratory and requires prospective external validation before any forensic or clinical application.

## 1. Introduction

The identification and assessment of interpersonal physical violence constitute major challenges in forensic medicine and are of particular relevance to authorities investigating offences against life and bodily integrity. Furthermore, physical violence frequently co-occurs with other forms of abuse, including sexual, psychological, and economic violence [1]. Institutions and professionals within the social protection system, including social workers, the Police, and public prosecution services, play a central role in detecting and preventing violence. Nevertheless, dependence on these mechanisms may hinder the timely recognition of abuse and the implementation of effective preventive measures. Currently, the assessment of interpersonal physical violence relies primarily on the victim’s account and a forensic medical examination [2]. The complex nature of the relationship between victims and perpetrators, often influenced by psychological, social, or economic dependence, may discourage victims from disclosing their experiences or lead them to minimise or rationalise the perpetrator’s behaviour [3]. This issue is particularly salient in cases involving individuals in close personal relationships, especially in the context of domestic violence (DV) and intimate partner violence (IPV) [4,5]. Enhancing the objectivity of the assessment of interpersonal violence, therefore, remains a major challenge in forensic medicine. Any diagnostic tool developed for this purpose should be readily accessible and suitable for practical use by professionals and institutions involved in detecting violence.

One promising approach is the application of biochemical markers. From this perspective, interpersonal violence may be conceptualised as a potent stressor capable of triggering a cascade of physiological responses that support adaptation to an acute threat [6]. These processes involve activation of the autonomic nervous system, together with a range of endocrine changes. A central component of the physiological reaction to stress is the hypothalamic–pituitary–adrenal (HPA) axis [7]. Within this axis, cortisol, a steroid hormone secreted by the adrenal cortex, serves as a crucial hormonal mediator [8,9]. Cortisol exerts wide-ranging effects throughout the body, regulating carbohydrate metabolism, immune function, and inflammatory processes. It also plays a well-established role in modulating nervous system activity and adaptation to stress [10]. This hormone drives several pathophysiological changes triggered by traumatic stressors, including physical violence. Its secretion rapidly mobilises energy reserves and modulates immune activity when homeostasis is threatened.

As the unbound fraction readily diffuses from blood into saliva, salivary concentrations can be measured [11]. A major practical advantage of saliva as a diagnostic medium is that it can be collected easily and without invasive procedures. Moreover, salivary biomarker analysis is increasingly being incorporated into clinical practice for disease diagnosis and monitoring [12,13]. When interpreting cortisol concentrations in victims of interpersonal physical violence, the hormone’s circadian rhythm must be carefully considered. Levels are generally highest in the early morning, with a further increase following awakening known as the cortisol awakening response (CAR), and subsequently decline throughout the day to reach their lowest values during the evening and night [14,15]. Emerging evidence suggests that salivary biochemical markers may have practical value in assessing interpersonal violence. In particular, alterations in salivary cortisol have attracted particular interest as a potential indicator of exposure to abuse [16].

Despite evidence linking interpersonal violence with HPA-axis dysregulation, previous studies have predominantly focused on group-level differences in cortisol secretion rather than evaluating its diagnostic accuracy at the individual level. Consequently, evidence regarding clinically or forensically interpretable thresholds, sensitivity, specificity, and discriminatory performance remains scarce. Therefore, this preliminary pilot case–control study aimed to assess alterations in salivary cortisol concentrations among individuals exposed to recent interpersonal physical violence and to evaluate whether their preliminary measurement could support possible victim identification.

## 2. Materials and Methods

### 2.1. Participants

Participants in the study group were recruited from individuals referred for forensic medical examination at the Department of Forensic Medicine, Poznan University of Medical Sciences. All participants provided written informed consent before enrolment. Stringent eligibility criteria were applied. Only adults were included, with both women and men eligible to participate. Recruitment was restricted to individuals referred by local police units, thereby providing independent confirmation that the alleged exposure to violence was subject to an ongoing investigation. Participants were eligible only if the forensic examination was conducted within 48 h of the incident. To minimise the influence of the circadian rhythm of cortisol secretion, recruitment was limited to individuals examined between 8:30 and 10:30 a.m. Before study procedures were initiated, participants were informed about the project’s aims and underwent a detailed medical history assessment to verify eligibility for the exclusion criteria. Individuals receiving daily corticosteroid therapy for underlying medical conditions, as well as those with endocrine or autoimmune disorders, were excluded. Oral health was assessed during the initial examination, and individuals with unsatisfactory oral hygiene were ineligible to participate. Additional exclusion criteria included chronic occupational exposure to stress or violence, such as that experienced by military personnel, police officers, and members of other uniformed services. The control group was recruited concurrently from healthy volunteers, age-, gender- and BMI-frequency-matched. The study was conducted between March 2026 and June 2026 and was approved by the institutional Bioethics Committee (approval no. 311/25; 8 May 2025).

Given the preliminary and exploratory nature of the SALIVA (Saliva Analysis in Interpersonal Violence Assessment) project and the specific recruitment setting involving individuals undergoing forensic examination shortly after violent incidents, no formal a priori sample-size calculation was performed. The sample size was determined by the number of eligible participants recruited during the predefined study period.

### 2.2. Interview and Examination

Participants who satisfied the eligibility criteria underwent a comprehensive medical history assessment and physical examination. Detailed information regarding the circumstances of the incident was collected using a standardised form. The medical history included chronic conditions, regular medications, smoking status, and the time elapsed since the last oral intake. During the physical examination, height, body weight, blood pressure, and heart rate were recorded, and the oral cavity and mucous membranes were evaluated. Incident-related data included the event date and location and the relationship between the victim and the perpetrator. Cases were categorised as domestic violence (DV), intimate partner violence (IPV), urban violence (UV), or other. A formal forensic medical examination was then performed to document external injuries, and all available medical records related to the incident were reviewed. Any injuries to the oral mucosa or dental trauma that could cause contamination of the collected material with blood were excluded. Where injuries were identified, their severity was classified as minor, moderate, or severe in accordance with Articles 156 and 157 of the Polish Criminal Code and established forensic medical practice in Poland [17,18]. In cases where no visible external injuries were found, despite a confirmed incident of physical violence, they were defined as a violation of physical integrity in accordance with Article 217 of the Polish Criminal Code. The injuries and circumstances of the incident were also evaluated to determine whether they had posed an immediate risk of death or grievous bodily harm, as defined in Article 160 of the Polish Criminal Code [19]. Participants in the control group underwent the same medical history assessment and physical examination protocol.

### 2.3. Sample Collection

Saliva was collected using the passive drool method. Before collection, the procedure was explained to each participant by a physician to ensure correct sampling. Participants were instructed to lean forward and expectorate into sterile, graduated 50 mL Falcon tubes over a 10 min period. All samples were collected between 8:30 a.m. and 10:30 a.m., at least 1 h after the last oral intake to minimise potential food-related interference. Sampling was initiated no earlier than 15 min after the beginning of the forensic medical examination, allowing participants time to acclimatise to the examination setting and thereby reducing the potential influence of examination-related psychological stress. Before collection, participants were instructed to rinse their mouths to minimise the risk of sample contamination. Oral rinsing was conducted using a moderate amount of water, permitting the patient to rinse freely. A minimum sample volume of 0.5 mL was required. Samples visibly contaminated with blood or food debris were excluded from further analysis.

### 2.4. Sample Preparation and Analysis

Following collection, saliva samples were temporarily stored at 4 °C in a dedicated refrigerator and then transferred to the laboratory of the Department of Forensic Medicine at Poznan University of Medical Sciences for further processing. Samples were centrifuged at 3000× *g* for 20 min at 4 °C, and the resulting supernatant was used for analysis. Aliquots were stored at −70 °C until cortisol measurement. Salivary cortisol concentrations were determined using a commercially available Cortisol ELISA Kit (Neogen, Lansing, MI, USA, SKU No. 402710), in accordance with the manufacturer’s instructions. This assay was based on the competitive ELISA principle. Microplate wells were pre-coated with anti-cortisol antibodies. Standards, controls, and samples were added and allowed to bind to the immobilized antibodies. Next, the diluted enzyme conjugate was added and the mixture was shaken and incubated at room temperature. During the incubation, competition for binding sites was taking place. After incubation and washing, a chromogenic substrate was introduced, resulting in a color reaction inversely proportional to the amount of analyte present in the sample. The sample volume used for analysis was 10 µL, diluted with 990 µL of extraction buffer. Final dilution factor was 100. The absorbance was measured at 650 nm. Sample concentrations were calculated using a standard calibration curve (Standard curve R^2^ ≥ 0.98). Analytical range was from 0.1 to 10 ng/mL. Quantification limit was 0.04 ng/mL. Intra-assay was 8.8%. Inter-assay was 8.8%. Cross reactivity for cortisol was 100%. All samples were duplicates. Laboratory personnel were blinded to group allocations. Samples were anonymous. The workflow for recruiting the study group, collecting samples, and processing them is shown in Figure 1.

### 2.5. Diagnostic Test Framework

Salivary cortisol concentration measured by ELISA was considered the index test. Exposure status served as the reference classification and was established independently of the cortisol result. Participants in the violence-exposed group had been referred by the Police for forensic medical examination following a reported incident of interpersonal physical violence and underwent standardised forensic evaluation, including medical history, physical examination, documentation of injuries, and review of available incident-related medical records. Control participants had no reported recent exposure to interpersonal physical violence. Cortisol results were not used to establish exposure status. The reporting of this preliminary diagnostic accuracy study was guided, where applicable, by the Standards for Reporting Diagnostic Accuracy Studies (STARD) recommendations.

Primary outcome was difference in morning salivary cortisol concentration between violence-exposed participants and controls. Secondary/exploratory outcome was apparent discriminatory performance of salivary cortisol assessed using ROC analysis.

### 2.6. Statistical Analysis

Continuous variables were summarised as means and standard deviations (SD). The normality of data distribution was assessed using the Shapiro–Wilk test. As the analysed variables showed distributions consistent with normality, the study and control groups were compared using the independent-samples Student’s *t*-test. Comparisons of cortisol concentrations according to the type of violence were performed using the Kruskal–Wallis test due to the lack of normality within the small subgroups. The association between cortisol concentrations and the time elapsed since the violent incident was assessed using Spearman’s rank correlation coefficient.

To assess whether the association between exposure to violence and salivary cortisol levels was independent of potential confounding factors, a general linear model (GLM) was performed. Salivary cortisol level was included as the dependent variable, while group (study vs. control), age, sex, body mass index (BMI), and sample collection time were entered as independent variables. Group and sex were included as categorical factors, whereas age, BMI, and sample collection time were entered as continuous covariates. Regression coefficients (β) with standard errors and 95% confidence intervals (95% CIs) were reported.

The distributions of cortisol concentrations in the control and study groups, as well as across different types of violence (assault, domestic violence [DV], intimate partner violence [IPV], and other), were visualised using violin plots with superimposed box plots and individual observations. The relationship between the time interval and cortisol concentration in the study group was additionally explored using a scatter plot with locally estimated scatterplot smoothing (LOESS) to visualize potential nonlinear trends.

Receiver operating characteristic (ROC) curve analysis was performed to evaluate the ability of salivary cortisol levels to discriminate between victims of violence and matched controls. Discriminatory performance was expressed as the area under the ROC curve (AUC) with a 95% CI. The optimal cut-off value was determined based on the maximum Youden index, and the corresponding sensitivity and specificity with 95% CIs were calculated. Bootstrap resampling with 1000 replicates was used to estimate the uncertainty of ROC-derived parameters and the 95% CI for the optimal cut-off value.

Statistical significance was set at alfa = 0.05. Data were analysed using Statistica 13.3 software (StatSoft, Cracow, Poland), and MedCalc 23.4.0 software (Ostend, Belgium).

No artificial intelligence tools were used for literature screening, study selection, data extraction, scientific content generation, evidence synthesis, interpretation, or any methodological or scientific decision-making. Artificial intelligence tools were used exclusively for post-writing linguistic refinement (grammar and stylistic editing), as disclosed in the Acknowledgements.

## 3. Results

### 3.1. Comparison of the Study and Control Groups

The study group comprised 26 victims of violence and was compared with a control group of 26 individuals. When assessing the characteristics of violent incidents in the study group, 38.5% experienced intimate partner violence (IPV), 30.8% assaults, 19.2% domestic violence (DV), and 11.8% were categorised as other types of incidents. In most cases, victims suffered minor injuries, consistent with the categories defined in the Polish Criminal Code (73.1%), while moderate injuries were observed in 15.4% of victims. In 11.5% of cases, only violation of physical integrity was experienced, with no visible signs of bodily injury. No severe injuries were observed in the study group. Only in two cases was it found that the victim was exposed to immediate danger of loss of life or a grievous bodily injury, in the circumstances of the violent incident.

As expected from the matching procedure, there was no significant difference in age between the groups (38.46 ± 14.83 vs. 37.54 ± 14.53 years, respectively; *p* = 0.822). The groups were also comparable in terms of BMI (23.34 ± 5.07 vs. 24.62 ± 4.49 kg/m^2^; *p* = 0.338).

In contrast, significant differences were observed in cardiovascular parameters. The study group had significantly higher systolic blood pressure (139 ± 14 vs. 122 ± 10 mmHg; *p* < 0.001), diastolic blood pressure (86 ± 11 vs. 77 ± 7 mmHg; *p* = 0.002), and pulse rate (85 ± 11 vs. 76 ± 10 beats/min; *p* = 0.005) compared with the control group.

Most notably, salivary cortisol levels were markedly higher in the study group than in the matched controls (110.33 ± 54.41 vs. 23.49 ± 6.29 ng/mL; *p* < 0.001), corresponding to an approximately 4.7-fold higher mean cortisol concentration among victims of violence (Figure 2).

Cortisol concentrations showed some variation according to the type of violence (Figure 3). The lowest concentrations were observed among victims of domestic violence (DV), whereas generally higher values were found in the assault, intimate partner violence (IPV), and other violence groups. Considerable within-group variability was evident, particularly in the assault and DV groups, while cortisol concentrations in the IPV and other violence groups tended to cluster at higher levels. However, these apparent differences in distribution were not statistically significant (*p* = 0.191), and the small subgroup sizes warrant cautious interpretation of these patterns.

The mean time from violent incident to saliva collection was 21.8 h (range: 8.5–43.5). No significant correlation was observed between salivary cortisol concentrations and the time elapsed between the violent incident and saliva collection (*p* = 0.699). The scatter plot illustrates the relationship between the interval (hours) and cortisol concentration, with the smoothed curve showing the overall pattern of association (Figure 4). Considerable variability in cortisol concentrations was observed across the entire range of intervals. The smoothed trend suggested a weak nonlinear relationship: cortisol concentrations remained relatively stable or increased slightly up to approximately 25–30 h, followed by a decline at longer intervals. However, the wide dispersion of individual observations indicates that the time interval alone does not appear to strongly explain the variability in cortisol concentrations.

### 3.2. Multivariable Analysis of Salivary Cortisol Levels

In the general linear model adjusted for age, BMI, sample collection time, and sex, membership in the study group remained strongly and independently associated with higher salivary cortisol levels (β = 0.759, 95% CI: 0.557–0.960; *p* < 0.001). In contrast, age (β = 0.035, 95% CI: −0.180–0.249; *p* = 0.747), BMI (β = 0.030, 95% CI: −0.181–0.240; *p* = 0.778), sample collection time (β = −0.017, 95% CI: −0.226–0.193; *p* = 0.872), and female sex (β = −0.047, 95% CI: −0.247–0.153; *p* = 0.639) were not significantly associated with cortisol levels. These findings indicate that the substantially higher salivary cortisol levels observed among victims of violence were maintained after adjustment for the included potential confounding factors.

### 3.3. ROC Analysis of Salivary Cortisol

ROC analysis demonstrated excellent discriminatory ability of salivary cortisol levels for distinguishing victims of violence from matched controls, with an AUC of 0.991 (95% CI: 0.962–1.000; *p* < 0.0001)—Figure 5. The optimal cut-off value of >35.45 yielded a sensitivity of 92.31% (95% CI: 74.9–99.1%) and a specificity of 100% (95% CI: 86.8–100.0%). Bootstrap analysis confirmed the robustness of the estimated cut-off, with a 95% CI ranging from >32.17 to >35.45. Overall, salivary cortisol showed near-perfect apparent discrimination between the study and control groups in this sample.

## 4. Discussion

The analyses performed showed approximately 4.7-fold higher mean cortisol concentration among victims of physical violence compared to the control group not exposed to violence. Moreover, higher salivary cortisol levels observed in the study group were maintained after adjustment for potential confounding factors. Furthermore, a cut-off value of >35.45 ng/mL was obtained, keeping a sensitivity of 92.31% and a specificity of 100%, showing near-perfect apparent discrimination between the victims of physical interpersonal violence and the control group.

Salivary analysis is gaining increasing recognition across contemporary medical sciences. In clinical practice, it is being applied to rapid preliminary assessment, screening, and health monitoring in a wide range of conditions, including oncological, neurological, autoimmune, and other systemic diseases [20,21]. Its use is also expanding in forensic medicine, where salivary analysis is emerging as a valuable diagnostic approach [22]. Salivary testing enables the detection of xenobiotics, including psychoactive substances, and is used in rapid drug screening procedures conducted by police services [23]. The ease of sample collection and the relative stability of certain biomarkers are contributing to the growing importance of saliva in modern forensic science. Its principal advantage is the entirely non-invasive nature of collection, which allows samples to be obtained directly at the scene without the need to transport the individual to a medical facility. The procedure is rapid and straightforward, while the compact collection kits can be readily incorporated into the standard equipment of law enforcement and investigative services.

Molecular analytical techniques have become increasingly important in contemporary forensic science. Current research highlights the expanding application of biomarkers in forensic investigations [24]. Their incorporation into diagnostic and evidentiary workflows is intended primarily to enhance objectivity, particularly in cases where testimonial evidence requires independent corroboration. By reflecting biological changes induced by a specific event or exposure, biomarkers may provide measurable evidence to reconstruct and verify relevant circumstances. Cortisol is among the biomarkers that have attracted particular interest in forensic practice [25,26]. This steroid hormone exhibits a pronounced circadian pattern of secretion and plays an important role in regulating the body’s adaptation to stress, including psychosocial stressors [27,28,29]. Moreover, stress exerts widespread effects on the central nervous system, engaging neural circuits involved in cognitive processing, reward, executive control, fear, and anger [30]. These processes engage the hypothalamus, promoting the release of corticotropin-releasing hormone, which in turn stimulates the secretion of adrenocorticotropic hormone from the anterior pituitary. Adrenocorticotropic hormone then acts as a principal regulator of cortisol synthesis and release [31]. Acute stress is typically associated with a marked increase in cortisol secretion, whereas prolonged stress exposure may alter basal secretory patterns. These changes have been described as occurring in two successive stages. The initial hypercortisolaemic phase is characterised by sustained activation of the HPA axis, elevated cortisol concentrations, and a blunted decline across the day. This may subsequently transition into a hypocortisolaemic phase, marked by a flatter diurnal cortisol profile, attenuation of the cortisol awakening response, and reduced variation in concentrations throughout the day [29,30,31,32]. Recent studies have suggested that serum cortisol measurement may help distinguish between consensual and non-consensual sexual activity. In one study, serum cortisol concentrations were 1.1- to 3.1-fold higher in survivors of sexual assault than in controls who had engaged in consensual sexual intercourse, indicating a substantial increase in circulating cortisol following assault [33]. In that study, cortisol was quantified in blood samples, the collection of which requires trained medical personnel and is generally performed in a healthcare setting. This requirement limits the method’s feasibility in the immediate aftermath of an incident, particularly at the scene or when a victim first reports to a police station.

Salivary cortisol may serve as a potential biomarker of acute stress associated with reported interpersonal violence, reflecting the physiological response to traumatic experiences. Salivary cortisol concentrations reflect circulating free cortisol because the unbound fraction of the hormone readily diffuses from blood into saliva [34]. When analysing alterations in salivary cortisol, they should also be interpreted in the context of the normal range. Furthermore, variations in concentration ranges across time of day must be taken into account due to the circadian rhythm of cortisol secretion. Extant literature posits that in healthy adult populations, salivary cortisol concentrations exhibit a distinct diurnal rhythm, peaking significantly in the early morning. Alterations in salivary cortisol should be interpreted against appropriate reference values, with particular consideration given to variation across the day arising from its circadian secretion pattern. In healthy adults, salivary cortisol typically follows a distinct diurnal profile, peaking in the early morning. Reported morning values range from approximately 10 to 27 nmol/L, with a mean concentration of 15.5 nmol/L. Levels then decline throughout the day, reaching an evening nadir of approximately 2–4 nmol/mL, with a reported mean of 3.9 nmol/L. These ranges may also differ according to sex [35]. In another study of healthy participants, salivary cortisol measured between 10:00 a.m. and 12:00 p.m. had a mean value of 5.7 ng/mL. Considerable inter-individual variability was observed, as indicated by a standard deviation of 6.35 ng/mL [36]. A study evaluating a second-generation monoclonal antibody-based assay established salivary cortisol reference intervals of 1.50–22.02 nmol/L at awakening, 1.50–20.87 nmol/L one hour after awakening, 1.50–12.51 nmol/L at noon, 1.50–13.03 nmol/L at 4:00 p.m., 1.50–9.52 nmol/L at 8:00 p.m., and 1.50–6.28 nmol/L at midnight [37].

Available evidence suggests that cortisol measurement may contribute to the assessment of interpersonal violence, particularly in the context of intimate partner violence. In a Spanish study involving women exposed to IPV, elevated evening salivary cortisol values were observed more frequently than in the control group. Moreover, evening concentrations in women with a history of IPV exceeded the normative thresholds defined in the CIRCORT database [38]. A further Spanish study reported altered salivary cortisol profiles in women exposed to IPV. Among these participants, those with depressive symptoms exhibited lower morning cortisol concentrations than women without such symptoms, as well as higher evening concentrations than controls [39]. This pattern may reflect prolonged exposure to IPV, sufficient to contribute to the development of depressive symptoms and a hypocortisolaemic profile characterised by flatter diurnal cortisol slopes. Consistent findings were reported in a separate study of women exposed to IPV, which demonstrated elevated salivary cortisol concentrations and suggested that physical violence may exert a greater influence on cortisol regulation than psychological violence [40]. The differential effects of specific forms of violence on salivary cortisol were also examined in a Portuguese study involving women exposed to IPV. An absent cortisol awakening response was associated with physical violence, while psychological violence was linked to alterations in morning salivary cortisol concentrations. The findings further suggested that prolonged and severe exposure to violence may disrupt HPA-axis regulation in women experiencing IPV [41]. In a Cameroonian cohort, mothers exposed to IPV exhibited higher morning cortisol concentrations and greater overall cortisol output than women in the control group [42]. Similarly, research involving men exposed to IPV showed that greater victimisation was associated with higher midday cortisol concentrations [43].

Interestingly, salivary cortisol concentrations were not significantly associated with the time elapsed between the violent incident and sample collection within the predefined 48 h recruitment window. Although this finding may suggest that the observed cortisol elevation was not strongly dependent on sampling delay within this interval, it should be interpreted cautiously, given the small sample size and the dynamic nature of the HPA-axis response to acute stress. Serial sampling in larger cohorts is required to characterise the temporal profile of salivary cortisol following interpersonal physical violence.

The non-invasive nature of saliva collection, together with its simplicity and suitability for repeated sampling, makes salivary cortisol an attractive candidate for further investigation as an adjunctive acute stress biomarker for assessing individuals with suspected recent exposure to interpersonal violence. Rather than serving as a stand-alone diagnostic test, cortisol measurement may ultimately prove most useful when interpreted alongside forensic examination, incident-related information, and potentially other biochemical markers. Future research should also include other factors that may influence salivary cortisol levels, such as alcohol and recreational drug use, psychiatric conditions, pain, sleep deprivation, physical activity, physical illness, fear and anxiety levels, menstrual cycle phase, oral contraceptive use, menopausal status, anticipation in legal procedures, occupational stress, and other acute stressors.

### Study Limitations

The major limitation of this study is its small sample size. Nevertheless, the investigation forms part of the broader SALIVA project, which is intended to further evaluate salivary biochemical markers in individuals exposed to violence and is currently at a preliminary stage. Another limitation resulting from the sample size is the limited ability to compare subgroups due to the high heterogeneity within small groups. An additional limitation is the reliance on a single salivary cortisol measurement, which precluded assessment of the participants’ full diurnal secretion profile. Obtaining multiple samples was not feasible due to the procedural constraints of the ongoing investigations. Furthermore, the diagnostic threshold was derived and evaluated on the same relatively small dataset, which may yield optimistic estimates of discriminatory performance. Although bootstrap resampling was used to quantify the uncertainty of the ROC-derived estimates, this procedure does not substitute for external validation of the proposed threshold.

Nevertheless, the observed discriminatory performance should not be interpreted as direct evidence of diagnostic specificity for interpersonal violence. Cortisol is a dynamic physiological marker influenced by multiple biological, psychological, and environmental factors, and its interpretation therefore requires consideration of potential confounders and the temporal context of sample collection. Also, possible circadian and behavioural confounders, particularly awakening time and the interval between the event and sampling, should be considered during result interpretation. Therefore, the results indicate that individuals referred for forensic examination after reported violence exhibit substantially higher cortisol levels. Nevertheless, these data do not yet establish that cortisol serves as a standalone specific biomarker for identifying victims of violence. The use of healthy volunteers as controls may have increased the observed separation between groups and does not reproduce the full spectrum of alternative acute stressors encountered in forensic or emergency settings. Future diagnostic studies should therefore include clinically relevant comparator groups, particularly individuals exposed to acute psychological stress or accidental physical trauma unrelated to interpersonal violence.

Participants were recruited from police-referred forensic examinations. Consequently, exposure status was determined on the basis of reported violence, Police initial investigation and medico-legal assessment rather than definitive judicial adjudication. This should not necessarily be considered an omission, as definitive judicial confirmation is rarely available in forensic research. Nevertheless, some degree of exposure misclassification cannot be excluded. Importantly, such misclassification would be expected to attenuate the observed differences between groups if individuals who were not true victims were incorrectly classified as exposed. Despite this potential source of bias, the study demonstrated substantial between-group differences, suggesting that the observed associations are unlikely to be explained solely by exposure misclassification.

## 5. Conclusions

This preliminary study provides proof-of-concept evidence that salivary cortisol concentrations are markedly elevated in individuals examined shortly after exposure to interpersonal physical violence compared with matched healthy controls. This association remained significant after adjustment for the included potential confounding factors, supporting the biological relevance of acute HPA-axis activation and the feasibility of salivary cortisol research in this forensic setting. Furthermore, salivary cortisol demonstrated near-perfect apparent discrimination between violence-exposed individuals and controls, indicating its potential value as an objective, non-invasive biomarker of recent acute stress associated with interpersonal physical violence.

However, the observed discriminatory performance should not be interpreted as evidence that salivary cortisol is specific for interpersonal violence. Cortisol is a dynamic stress-related biomarker influenced by numerous biological, psychological, and environmental factors, and the diagnostic threshold identified in the present cohort was derived and evaluated within the same relatively small sample. Accordingly, the proposed cut-off should be interpreted cautiously and regarded as exploratory rather than as a clinically or forensically validated diagnostic threshold.

Future studies should validate these findings prospectively in larger and more heterogeneous populations and include clinically relevant comparator groups exposed to other acute stressors or accidental physical trauma. Although no significant association between salivary cortisol concentrations and the time elapsed since the violent incident was observed within the 48 h sampling window in the present cohort, serial sampling in larger populations is needed to characterise the temporal cortisol response following interpersonal physical violence more precisely. Independent external validation of the proposed diagnostic threshold will also be essential before any diagnostic or forensic application can be considered. Overall, the present findings provide proof-of-concept for further development of salivary cortisol as a component of objective biomarker-based approaches supporting the assessment of recent interpersonal physical violence.

## Figures and Tables

**Figure 1:**
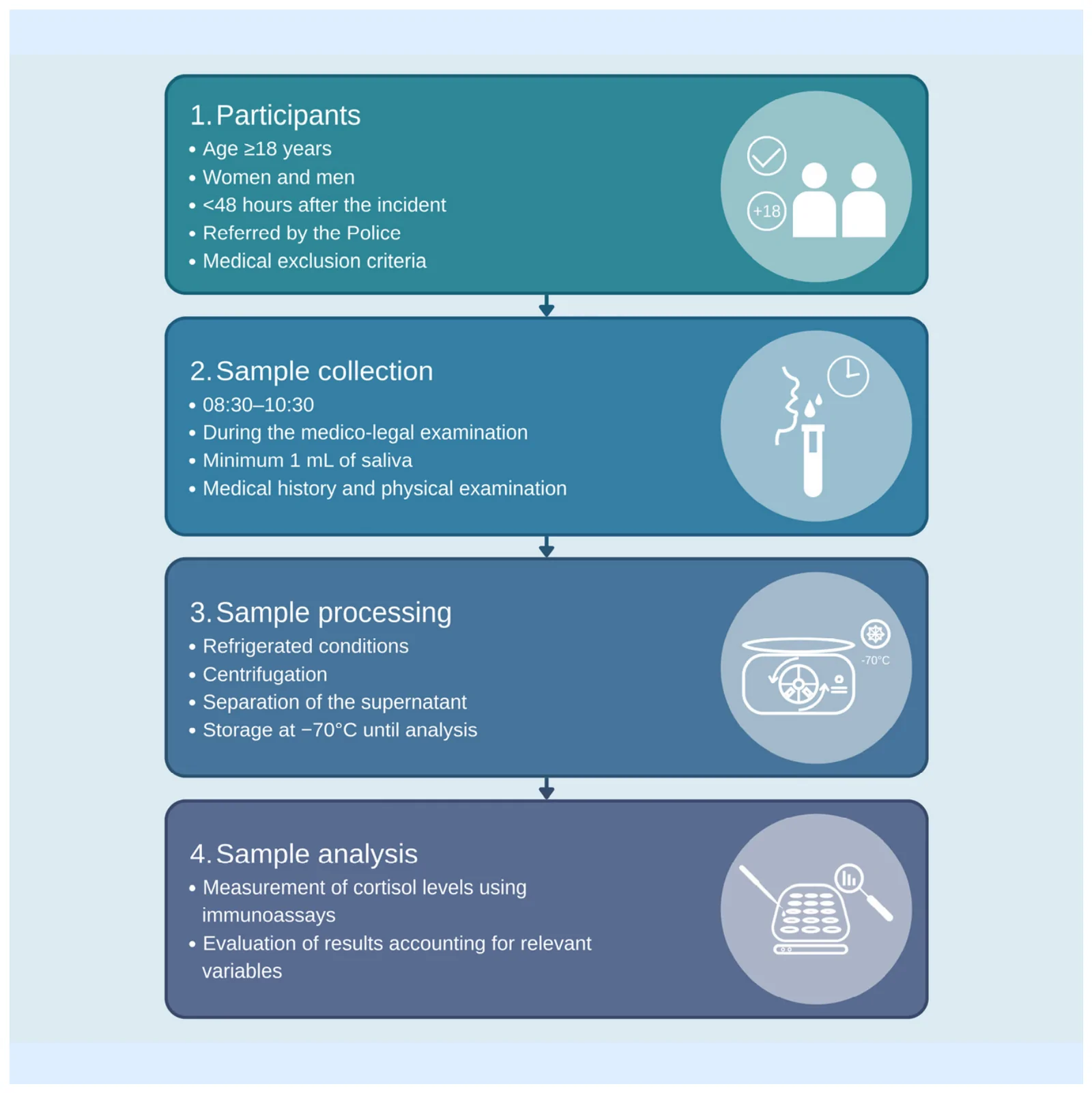
Procedural framework for participant recruitment, sample collection, and subsequent processing.

**Figure 2:**
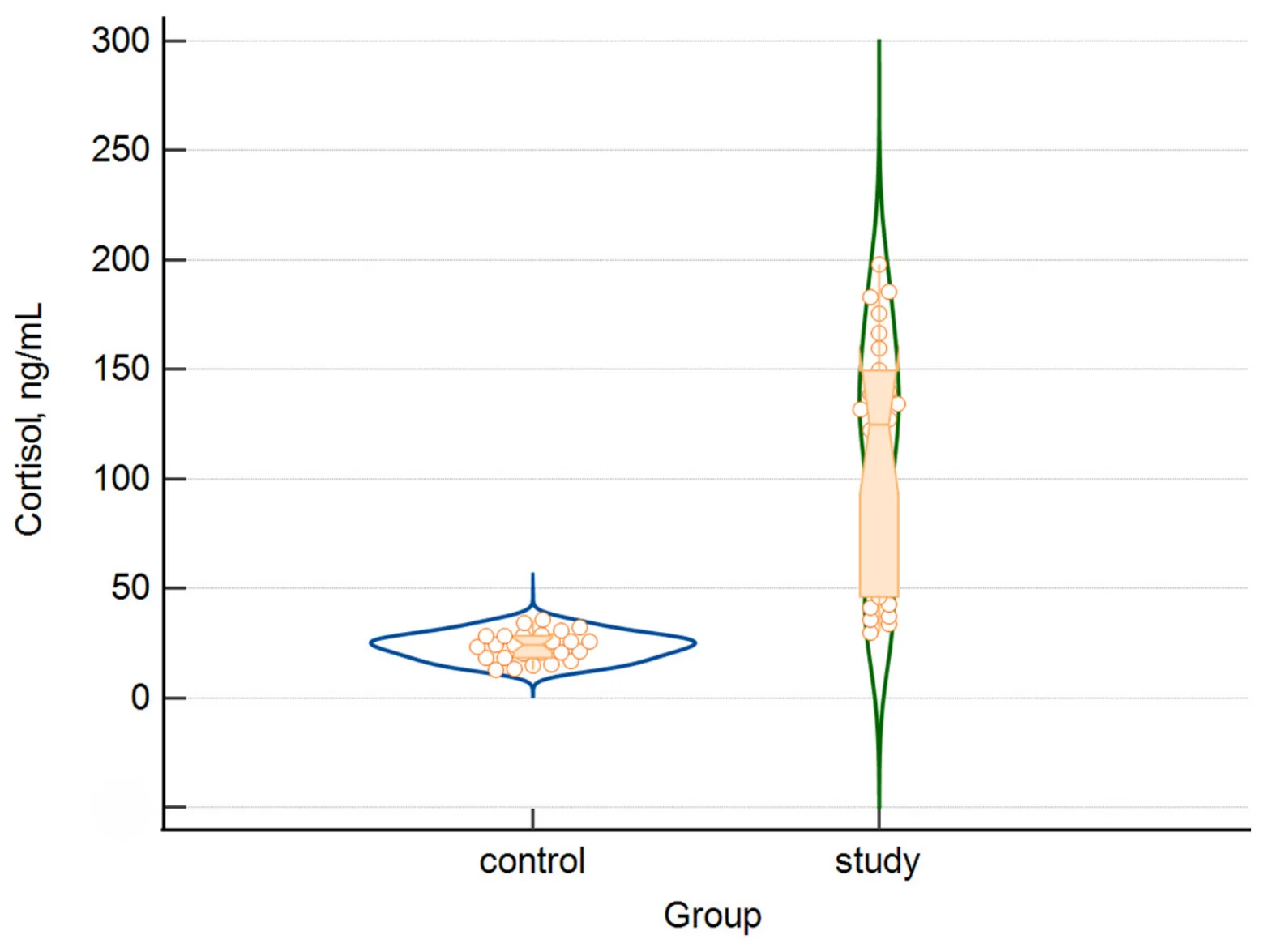
Violin plot presenting distribution of salivary cortisol between victims of violence and controls.

**Figure 3:**
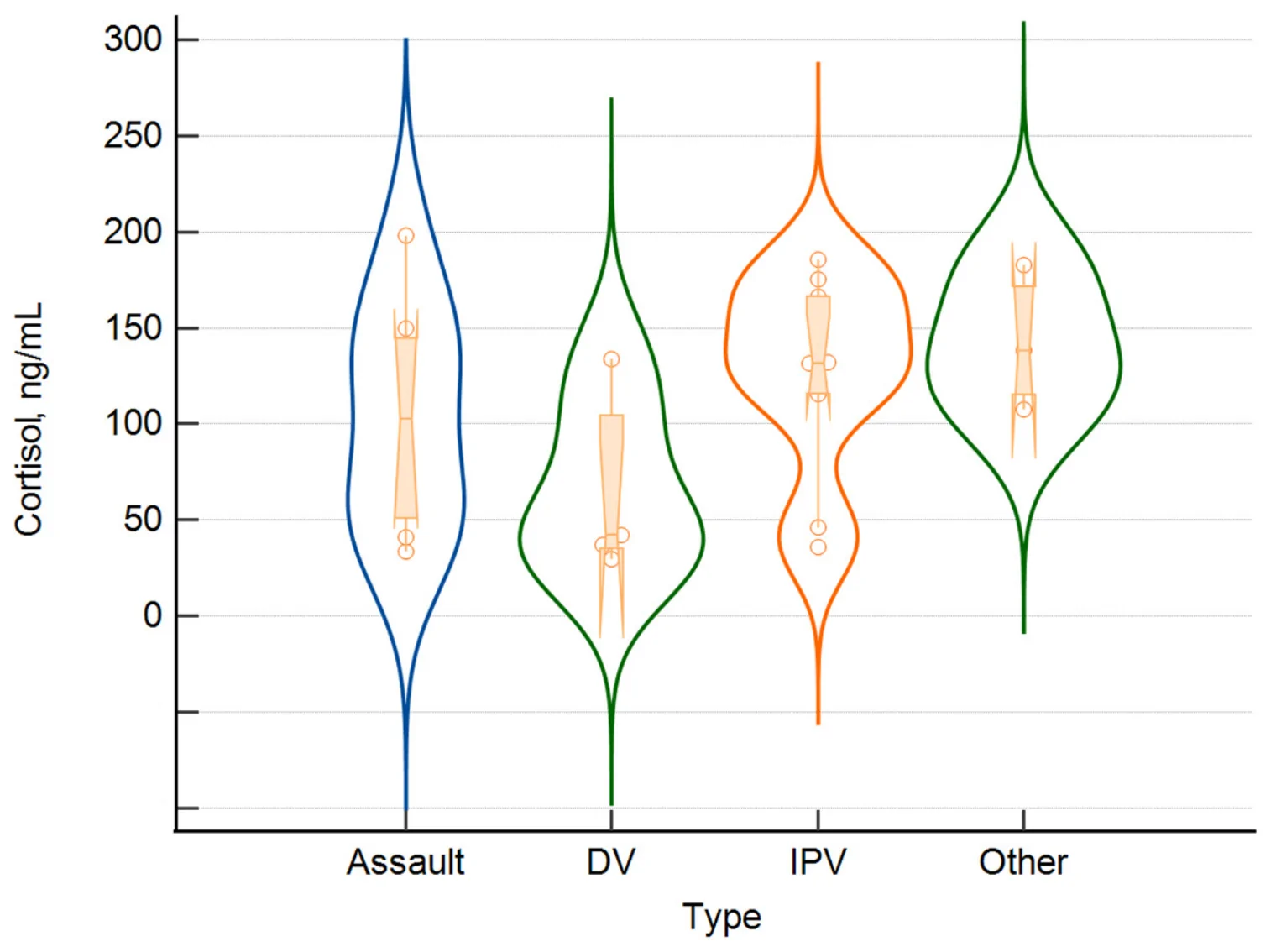
Violin plot presenting distribution of salivary cortisol according to the type of violence.

**Figure 4:**
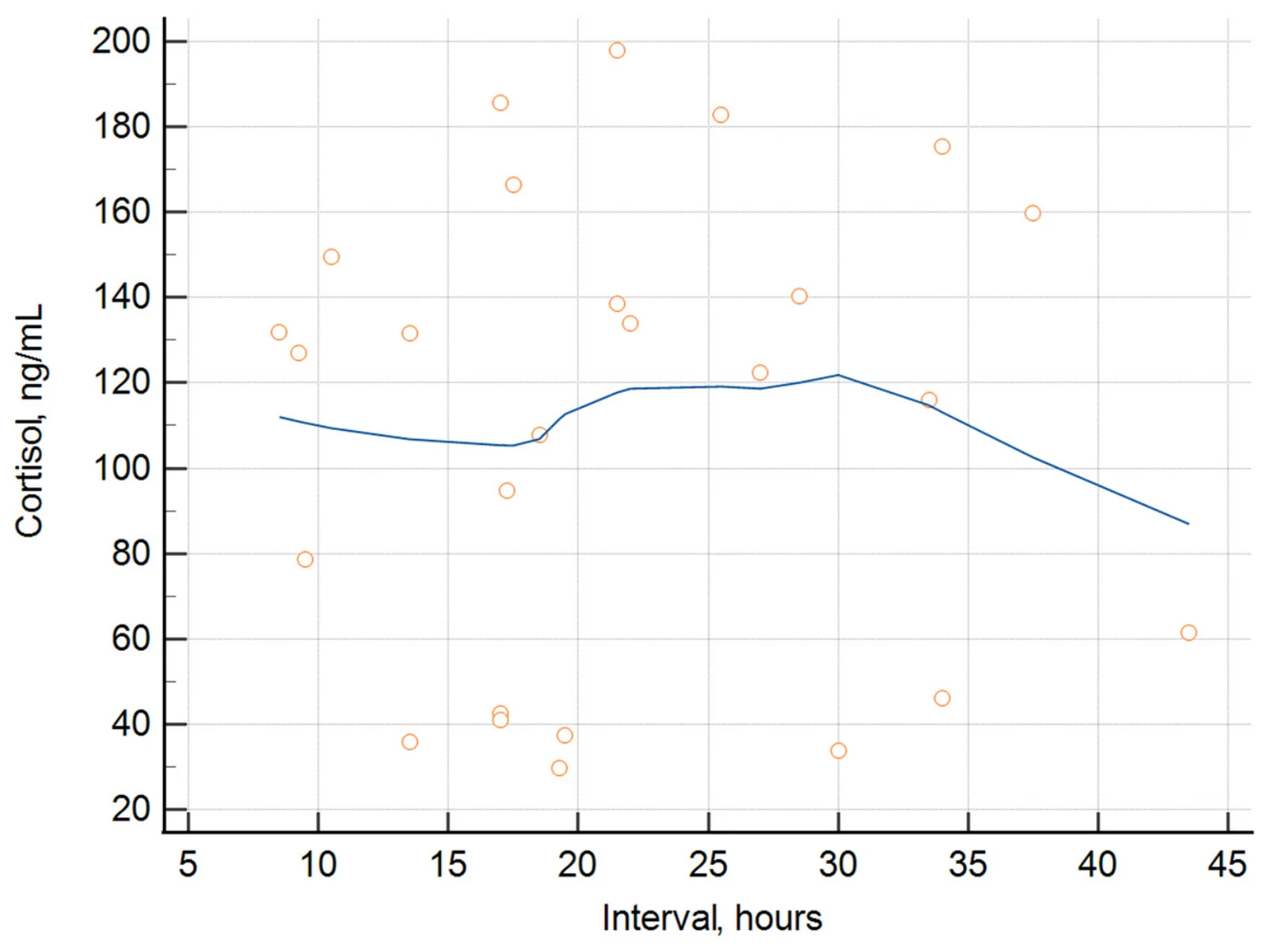
Relationship between the time interval and cortisol concentration in the study group.

**Figure 5:**
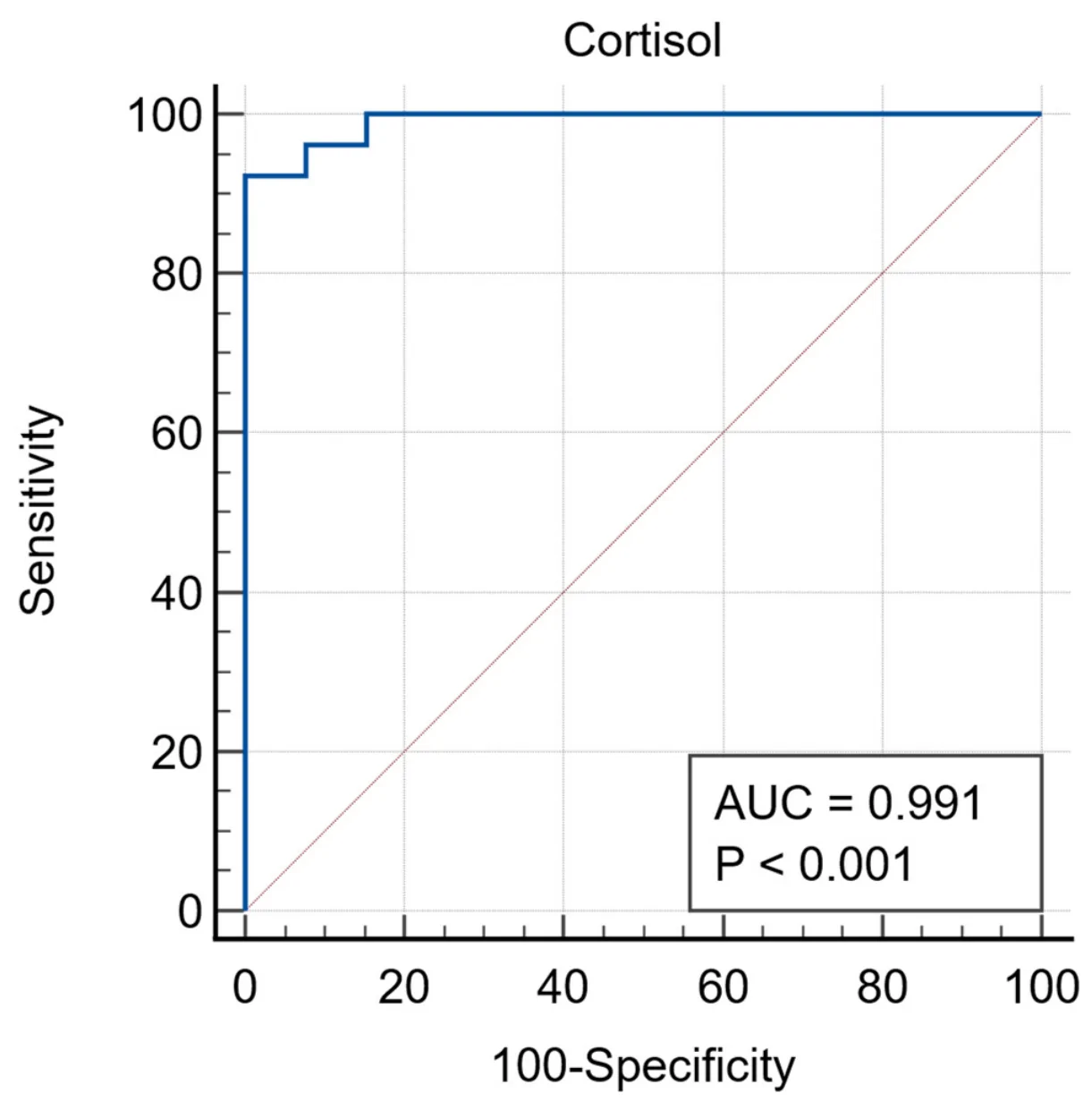
Receiver operating characteristic curve of salivary cortisol for discrimination between participants exposed to recent interpersonal physical violence and controls.

## Data Availability

Data are available upon request from the corresponding author.
